# From inflammation to malignancy: the dynamic evolution of cancer-associated fibroblasts in IBD-CRC

**DOI:** 10.1016/j.neo.2026.101310

**Published:** 2026-04-21

**Authors:** Nobuhiro Naito, Hiroaki Kasashima, Tatsunari Fukuoka, Yuki Nakanishi, Naoko Ohtani, Kiyoshi Maeda

**Affiliations:** a Graduate School of Medicine, Osaka Metropolitan University, Department of Gastroenterological Surgery, 〒545-8585, 1-4-3 Asahi-machi, Abeno-ku, Osaka-shi, Osaka, Japan; b Department of Gastroenterological Surgery, Osaka City General Hospital, 〒534-0021, 2-13-22 Miyakojimahondori, Miyakojima-ku, Osaka, Japan; c Graduate School of Medicine, Kyoto University, Department of Gastroenterology and Hepatology, 〒606-8507, 54 Shogoin-kawahara-cho, Sakyo-ku, Kyoto, Japan; d Graduate School of Medicine, Osaka Metropolitan University, Department of Pathophysiology, 〒545-0051, 1-5-7 Asahi-machi, Abeno-ku, Osaka-shi, Osaka, Japan

**Keywords:** IBD-CRC, Cancer-associated fibroblasts (CAFs), Tumor microenvironment, Metabolic reprogramming, Epigenetic memory, Therapeutic targeting

## Abstract

Inflammatory bowel disease–associated colorectal cancer (IBD-CRC) represents a distinct clinical and molecular entity characterized by long-standing inflammation and a unique "dysplasia-carcinoma" sequence. While epithelial genetic alterations are well-documented, the role of the mesenchymal stroma as a dynamic driver of malignant transformation remains underappreciated. In this review, we propose a conceptual framework where intestinal fibroblasts contribute significantly to niche construction as key components of the Tumor Microenvironment (TME) architecture. These cells are not merely passive scaffolds but dynamically co-evolve with the epithelium and immune compartment, transitioning into active orchestrators of the pre-malignant niche.

We highlight the emerging concept of "stromal inflammatory memory," which is hypothesized to be encoded through stable epigenetic imprinting and persistent metabolic reprogramming, characterized by a PFKFB3-mediated glycolytic shift. This metabolic priming sustains a pro-tumorigenic environment even during clinical remission. We further delineate how the loss of apical polarity regulators, such as aPKC, triggers a cascade of epithelial-stromal crosstalk involving chronic microbial sensing and hyaluronan-driven signaling. This co-evolution leads to the diversification of cancer-associated fibroblast (CAF) subsets, including inflammatory (iCAF), myofibroblastic (myCAF), and antigen-presenting (apCAF) populations. These subsets collectively orchestrate immune evasion via T-cell exhaustion and physical immune exclusion.

Finally, we discuss the clinical implications of the "Reverse Warburg Effect" and exosome-mediated chemoresistance. We emphasize that effective therapeutic strategies must transcend epithelial-centric models to target the stromal axis. By integrating recent advances in single-cell and spatial transcriptomics, we identify novel opportunities for "stromal reprogramming" through metabolic, epigenetic, and senomorphic interventions to prevent and treat IBD-CRC.

## Highlights

• Fibroblasts evolve from inflammation drivers to "TME Engineers" in IBD-CRC.
• Metabolic and epigenetic imprinting sustain a pro-tumorigenic stroma.
• Stromal senescence and matrix remodeling initiate the pre-malignant niche.
• CAF subsets orchestrate immune evasion via recruitment and signaling.
• Targeting "stromal memory" offers a novel paradigm for IBD-CRC therapy.

## Graphical abstract

**The Dynamic Evolution of Cancer-Associated Fibroblasts (CAFs) in IBD-CRC.** This diagram illustrates the phenotypic and functional transformation of intestinal fibroblasts during the progression from inflammatory bowel disease (IBD) to colitis-associated colorectal cancer (IBD-CRC). **Normal Homeostasis:** Quiescent fibroblasts support the intestinal stem cell (ISC) niche through the secretion of morphogens such as WNT, BMP, and R-spondin. **IBD Chronic Inflammation:** Persistent inflammation induces "Stromal Inflammatory Memory" via epigenetic imprinting and metabolic reprogramming. A hallmark of this stage is the PFKFB3-mediated glycolytic shift (Warburg-like effect), which sustains the activated inflammatory fibroblast (iCAF) state. **IBD-CRC Progression:** Chronic stromal priming culminates in the diversification of CAF subsets. Antigen-presenting CAFs (apCAFs) facilitate immune evasion by inducing T-cell exhaustion (TIM3+/LAG3+). Myofibroblastic CAFs (myCAFs) construct a "Rigid Niche" through excessive ECM deposition and LOXL2-mediated collagen cross-linking. Additionally, CAFs promote chemoresistance in tumor cells via the transfer of exosomal lncRNA H19. **Therapeutic Strategies:** Targeting this stromal axis through epigenetic modifiers, PFKFB3 inhibitors (PFK15), FAP-targeted CAR-T cells, or exosome inhibition represents a novel paradigm for IBD-CRC therapy.

**Figure:**
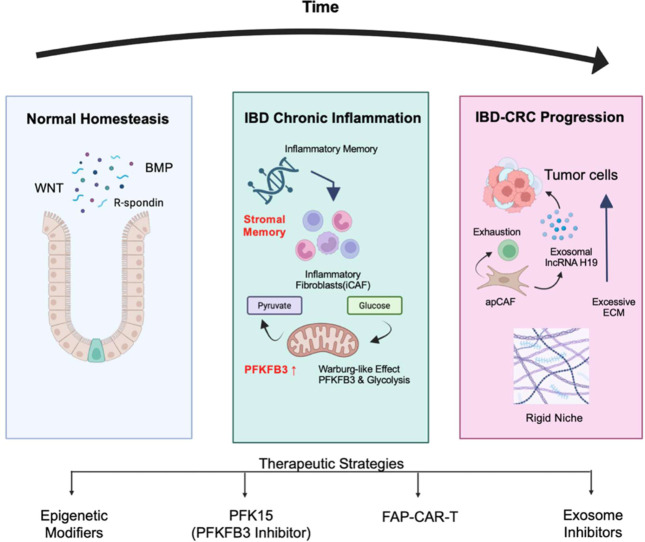

## Introduction

Chronic inflammation is a major contributing factor to the initiation and progression of malignancy, and its hallmark example, Inflammatory Bowel Disease (IBD) (Crohn's disease/Ulcerative Colitis), carries an inherent risk of developing IBD-Associated Colorectal Cancer (IBD-CRC) against a backdrop of chronic inflammation stemming from complex interplay among genetic, immunological, and environmental factors [[1], [2], [3]].

Unlike sporadic colorectal cancer, which typically follows the adenoma-carcinoma sequence, IBD-CRC progresses through a biologically distinct inflammation-dysplasia-carcinoma sequence. This process is characterized by a unique temporal and spatial pattern of genetic alterations. As highlighted by seminal studies [[1], [2], [3]], while sporadic CRC is almost always initiated by early loss of the *APC* tumor suppressor gene, IBD-CRC often exhibits *TP53* mutations at the very earliest stages—even in non-dysplastic, chronically inflamed mucosa. Conversely, *APC* mutations occur less frequently and typically much later in the transition to invasive carcinoma [3].

Furthermore, instead of arising from a single localized polyp, IBD-associated neoplasia often develops from a "field of injury," where long-standing oxidative stress and pro-inflammatory signaling induce widespread DNA damage and microsatellite instability across large areas of the colonic epithelium. This "field cancerization" creates a pre-malignant landscape that is uniquely shaped by the surrounding activated stroma, driving the progression from low-grade to high-grade dysplasia and ultimately to invasive adenocarcinoma.

The risk of IBD-CRC development increases in proportion to the duration of the disease. Since current therapeutic approaches targeting existing immune cells have limited efficacy in achieving mucosal healing or avoiding surgical intervention [[4], [5], [6], [7], [8]], the identification of novel therapeutic targets is an urgent necessity. Notably, recent studies have highlighted that mesenchymal stromal cells—particularly fibroblasts, which are essential for maintaining intestinal tissue homeostasis by producing the Extracellular Matrix (ECM)—become activated in an inflammatory environment and dynamically transform into functional subsets, acting as multifaceted regulators of the TME. While the term 'TME Engineers' captures their active role in tissue remodeling, it is essential to acknowledge the functional heterogeneity of fibroblast subsets and their complex, bidirectional interactions with the mucosal environment. They are now understood to be deeply involved in the chronic nature and fibrosis of IBD, contributing to the assembly of a TME that supports malignant transformation [9].

**Fig. 1:**
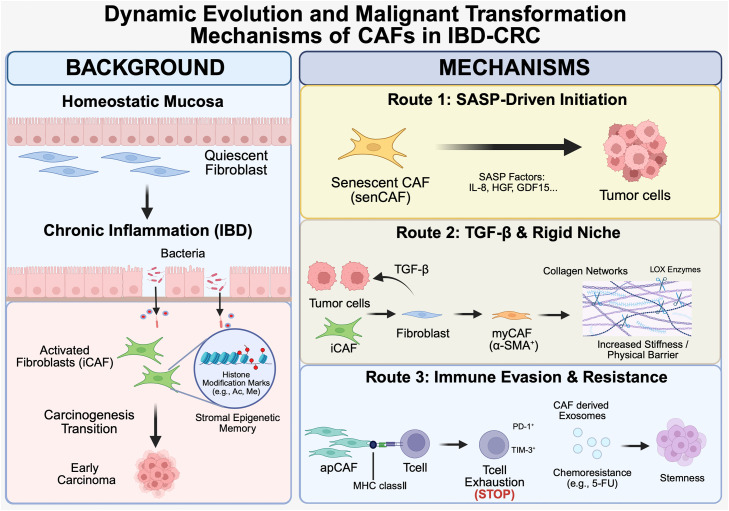
Dynamic stromal evolution and tumor-promoting mechanisms in IBD-CRC. The intestinal stromal niche undergoes a multi-step transformation during the inflammation-dysplasia-carcinoma sequence. (Left) Background: The Primed Stroma. Under Homeostatic conditions, quiescent fibroblasts maintain mucosal integrity. During Chronic Inflammation (IBD), persistent microbial stimuli and cytokines induce iCAFs(inflammatory CAFs). This phase is characterized by Stromal Epigenetic Memory (e.g., histone acetylation/methylation), which maintains a pro-tumorigenic niche even during clinical remission. (Right) Mechanisms: Key Drivers of Malignancy. Route 1 (SASP-Driven Initiation): senCAFs (senescent CAFs) secrete SASP factors (IL-8, HGF, GDF15), paracrinely promoting epithelial transformation and proliferation. Route 2 (TGF-β & Rigid Niche): Tumor-stroma interactions hyperactivate TGF-β signaling, driving myCAF (α-SMA^+^) differentiation. These cells secrete LOX enzymes to cross-link Collagen Networks, increasing tissue stiffness and creating a physical barrier against immune infiltration. Route 3 (Immune Evasion & Resistance): apCAFs (antigen-presenting CAFs) induce T cell exhaustionvia MHC class II-mediated cross-presentation (upregulating PD-1/TIM-3). Additionally, CAF-derived exosomes transfer lncRNA H19 to tumor cells, enhancing stemness and chemoresistance (e.g., 5-FU).

To provide clarity regarding the complex stromal landscape, it is essential to define the evolving terminology and functional diversity of fibroblast subtypes early in this discussion. Intestinal fibroblasts are no longer viewed as a uniform population but as a plastic and heterogeneous group of cells that adapt to their microenvironment [10,11]. Under homeostatic conditions, quiescent fibroblasts maintain structural integrity and support the intestinal stem cell niche. In the context of IBD, these cells transform into activated fibroblasts or myofibroblasts, characterized by the expression of α-smooth muscle actin (α-SMA) and a high capacity for extracellular matrix (ECM) production. As chronic inflammation progresses toward malignancy, these cells further diversify into distinct Cancer-Associated Fibroblast (CAF) subsets with specialized functions: (i) myofibroblastic CAFs (myCAFs), which drive desmoplasia and tissue stiffness primarily through TGF-β signaling; (ii) inflammatory CAFs (iCAFs), which promote a pro-tumorigenic niche by secreting cytokines such as IL-6 and CXCL12; and (iii) antigen-presenting CAFs (apCAFs), which express MHC class II molecules and contribute to immune evasion by inducing T-cell exhaustion. Understanding these functional transitions from homeostatic to pathogenic states is central to deciphering the unique "dysplasia-carcinoma" sequence in IBD-CRC(Fig. 1).

Building upon our laboratory's long-standing research on the functional and molecular characteristics of CAFs, this review aims to provide a comprehensive understanding of these stromal cells throughout the entire spectrum from inflammation to carcinogenesis, which is critically important for developing definitive new diagnostic and therapeutic strategies against IBD-CRC.

## Pathophysiological roles of fibroblasts in IBD

In the progression of IBD, gastrointestinal fibroblasts deviate from their function of maintaining physiological homeostasis and become actively involved in sustaining chronic inflammation, excessive fibrosis, and exacerbating tissue damage. This change is characterized by the activation of fibroblasts, which is triggered by the disease microenvironment rich in inflammatory cytokines and growth factors.

### Activation of fibroblasts in the inflammatory microenvironment

In the gut of IBD patients, there is an abundance of bacterial Toll-like receptor (TLR) ligands and numerous inflammatory cytokines, such as tumor necrosis factor-α(TNF-α), interleukin-1β(IL-1β), IL-6, and IL-17 [[12], [13], [14]]. These molecules bind to specific receptors on fibroblasts, thereby activating downstream signaling pathways including NF-κB, MAPK, and STAT3, which consequently lead to fundamental changes in fibroblast gene expression and function [15,16]. Specifically, TNF-α, a major therapeutic target in IBD, is known to induce the expression of adhesion molecules on fibroblasts, promoting their interaction with immune cells [17]. In response to these stimuli, activated fibroblasts differentiate into characteristic myofibroblasts. These cells exhibit increased expression of α-smooth muscle actin (α-SMA), acquiring contractility, and produce excessive extracellular matrix (ECM).

These myofibroblasts are a key factor in the intestinal tissue remodeling observed in IBD, particularly in the formation of fibrosis and strictures, and their presence strongly correlates with disease severity and the progression of fibrosis. Fibroblasts in the inflammatory microenvironment further complicate the dynamics of inflammation through interactions with immune cells. Additionally, IBD fibroblasts contribute to the persistence of inflammation by increasing the secretion of angiogenic factors, which promotes the recruitment of inflammatory cells to the site of inflammation [18]. The remodeling of the ECM creates a positive feedback loop where increased colonic tissue stiffness promotes further α-SMA and inflammatory gene expression, fostering the self-propagation of intestinal fibrosis [19]. This phenotypic transition is primarily driven by TGF-β signaling, which acts as a master switch for fibroblast activation. Recent studies demonstrate that the interaction between intestinal epithelial cells and the surrounding stroma hyperactivates both canonical Smad-dependent and non-canonical TAK1-mediated TGF-β pathways in fibroblasts [[20], [21], [22]]. This signaling not only induces α-SMA expression but also creates a feed-forward loop that sustains the activated state of myofibroblasts, even in the absence of continuous primary inflammatory stimuli. Furthermore, fibroblasts are known to promote inflammation via oxidative stress by overproducing lysyl oxidase (LOX) and LOXL1 [15]. Furthermore, the inflamed colonic mucosa often harbors hypoxic regions due to vascular disruption and increased metabolic demand. In such hypoxic environments, LOX expression is significantly upregulated, which has been shown to not only stiffen the ECM but also directly prime epithelial cells for transition toward a mesenchymal phenotype, even in the early stages of inflammation [23].

### Exacerbation of inflammation and crosstalk with immune cells

Under conditions of chronic inflammation, fibroblasts function not merely as passive scaffold cells but as active signaling hubs that sustain and amplify the inflammatory loop [24]. Particularly in IBD, recent studies have highlighted that bidirectional crosstalk between fibroblasts and immune cells plays a crucial role in the persistence of the pathology [4,5]. Upon activation in response to inflammatory cytokines such as TNF-α and IL-1β, fibroblasts themselves produce and secrete various inflammatory mediators [6]. These include chemokines (e.g., CCL2, CXCL1) that recruit immune cells to specific sites, and cytokines (e.g., IL-6, IL-11). These molecules continuously recruit inflammatory immune cells, such as macrophages, neutrophils, and monocytes, to the intestinal tissue, thereby amplifying the inflammatory response by sustaining immune cell infiltration.

Furthermore, fibroblasts directly influence the function of immune cells themselves. For example, it has been reported that activated fibroblasts in the gut of IBD patients secrete cytokines that modulate T cell proliferation and differentiation or directly interact with T cells by expressing antigen-presenting molecules (MHC Class II). Fibroblasts derived from Crohn's Disease (CD) patients have been shown to produce high levels of MMP10, which reduces the membrane-bound form of the immunosuppressive factor PD-L1, thereby activating the immune response [7]. Conversely, this phenomenon is not observed in Ulcerative Colitis (UC) patients, suggesting differences in fibroblast behavior depending on the disease type [7]. Furthermore, upon stimulation by IL-1β or TNF, certain fibroblast subsets produce the cytokine Oncostatin M (OSM), which forms a positive feedback loop by further promoting the activation of inflammatory cells [6]. Understanding this complex interaction between fibroblasts and immune cells is crucial for developing novel therapeutic strategies for IBD patients who exhibit resistance to conventional treatments targeting immune cells. Beyond direct fibroblast-immune cell interactions, the integrity of the epithelial barrier plays a foundational role in stromal priming. For instance, the loss of PKC λ/ι in the intestinal epithelium leads to a marked reduction in Paneth cell differentiation and the secretion of antimicrobial peptides, resulting in dysbiosis and a pro-inflammatory microenvironment that chronically activates resident fibroblasts [25]. Importantly, epithelial aPKC deficiency does not only impair the mucosal barrier but also actively remodels the stroma through the secretion of hyaluronan. This extracellular matrix component acts as a potent paracrine signal that activates resident fibroblasts, shifting them toward a pro-tumorigenic phenotype long before clinical malignancy is evident [26]. Moreover, the loss of epithelial aPKC (PKCλ/ι) leads to a profound defect in Paneth cell differentiation, resulting in an altered microbiome and increased exposure of the underlying stroma to bacterial components like lipopolysaccharide (LPS). This chronic microbial stimulation acts as a primary trigger for the initial activation of resident fibroblasts, establishing a 'pre-malignant' stromal state characterized by persistent TLR4 signaling long before the emergence of overt dysplasia [25].

In addition, recent clinical data have identified the OSM-OSMR signaling axis as a key mediator of resistance to anti-TNF therapy in patients with IBD (including both CD and UC) [6]. In this context, OSM-producing myeloid cells (such as inflammatory monocytes and macrophages) activate resident fibroblasts through OSMR, sustaining non-TNF-dependent inflammatory loops that prevent mucosal healing and contribute to the persistence of the pathogenic stroma. Furthermore, recent evidence highlights the role of Interleukin-34 (IL-34) as a critical mediator in the inflammatory stroma. In patients with IBD, particularly Crohn’s disease, IL-34 is significantly upregulated and acts on intestinal fibroblasts to stimulate the production of collagen and other profibrotic mediators, thereby contributing to tissue remodeling and stricture formation [27,28]. This cytokine establishes a potent link between myeloid cells and the mesenchymal compartment, further reinforcing the chronic inflammatory state.

### Metabolic reprogramming of fibroblasts: shift toward glycolysis

Recent studies have suggested that fibroblasts in IBD undergo significant metabolic reprogramming, characterized by a reciprocal shift toward glycolysis and a concomitant suppression of oxidative phosphorylation (OXPHOS) [8]. This metabolic profile resembles the "Warburg effect" observed in malignant cells, suggesting that fibroblasts in a chronic inflammatory niche may adopt a bioenergetic state that supports a pro-tumorigenic environment. A prominent feature of this transition is the upregulation of PFKFB3 (6-phosphofructo-2-kinase/fructose-2,6-bisphosphatase 3), a key regulator of glycolytic flux [8]. While PFKFB3-mediated metabolic shifts are hypothesized to sustain fibroblast activation and the secretion of pro-inflammatory cytokines, it remains to be fully determined whether this metabolic reprogramming is a primary driver of the dysplasia-carcinoma sequence or a secondary adaptation to the inflamed microenvironment.

Functional studies have demonstrated that pharmacological inhibition of PFKFB3 with PFK15 reduces inflammation and immune cell infiltration in murine colitis models [8]. It has been proposed that these metabolic alterations, including the transition away from OXPHOS, are part of an "inflammatory memory" potentially maintained through epigenetic modifications [29]. However, the exact molecular hierarchy and the causal link between this metabolic state and malignant transformation in human IBD-CRC require further clinical validation.

### Promotion of fibrosis

Intestinal fibrosis and subsequent stricture formation, which are long-term complications of IBD, primarily result from the overproduction and abnormal remodeling of the extracellular matrix (ECM) by fibroblasts residing in the intestinal interstitium [[30], [31], [32], [33], [34], [35], [36], [37]].

Specifically, fibroblasts activated by inflammation differentiate into myofibroblasts that express α-smooth muscle actin (α-SMA). These myofibroblasts excessively synthesize and secrete ECM proteins such as Type I and Type III collagen, fibronectin, and elastin, disrupting the balance of normal tissue repair [[33], [34], [35], [36], [37]] . This leads to the inappropriate accumulation of ECM, progressive tissue stiffening, functional impairment, and ultimately, the formation of intestinal strictures. It is crucial to distinguish between the fibrostenotic patterns of CD and UC. In CD, inflammation is typically transmural, leading to the activation of fibroblasts in the deeper muscularis propria and the formation of obstructive strictures. In contrast, UC-associated fibroblasts are primarily involved in mucosal and submucotic remodeling. While TGF-β signaling is a shared driver, recent evidence suggests that certain remodeling enzymes, such as MMP10, may show differential expression levels between CD and UC patients, potentially reflecting the distinct clinical manifestations of fibrosis in these two entities [7,24]. This complex fibrotic process involves a concerted effort of diverse signaling pathways. Among these, the Transforming Growth Factor-β(TGF-β)/Smad pathway is widely recognized to play a central role in promoting fibroblast activation and ECM production [30,31,38]. In addition to this, pathways such as the Wnt/β-catenin pathway, Notch signaling, and the MAPK pathway are also known to modulate the pro-fibrotic capabilities of fibroblasts. The aberrant activation of these pathways upregulates the expression of pro-fibrotic genes, directly leading to the excessive accumulation of ECM.

The role of fibroblasts in the inflammatory microenvironment is diverse. IBD fibroblasts exhibit increased capacity for proliferation and Type I collagen production compared to fibroblasts derived from healthy tissue [12] . Cadherin-11, a cell adhesion molecule between fibroblasts, promotes collagen synthesis in intestinal fibroblasts from Crohn's Disease (CD) patients [32]. In the structured intestines of CD patients, increased expression of genes encoding ECM components (collagen and fibronectin), ECM remodeling enzymes (MMP1, MMP2, MMP3, MMP7, MMP10, MMP13), and cytokines (IL-6, IL-1β, TGF-β, IL-11, IL-17, TNF-α, CXCL1, CXCL5) has been observed [[33], [34], [35], [36], [37]]. While excessive ECM deposition leads to fibrosis, the predominance of MMP activity over deposition results in tissue damage. Therefore, a balanced proteolytic activity and appropriate production of MMPs and their inhibitors (e.g., TIMPs) are essential for normal tissue repair [39]. Thus, fibroblasts in IBD play a central and multifaceted role in the complex pathogenesis, including the maintenance of chronic inflammation, the progression of fibrosis, and impaired tissue repair following damage.

It is important to note the clinico-pathological differences in fibroblast behavior between CD and UC. CD is characterized by transmural inflammation frequently leading to stenotic fibrosis, where a specific subset of "activated" fibroblasts in the deeper muscle layers undergoes myofibroblast transformation. In contrast, UC-associated fibroblasts are primarily localized in the mucosa and submucosa. Recent studies suggest that the expression of certain remodeling enzymes like MMP10 may differ between these two entities [7]. However, distinct CAF subpopulation profiles unique to CD-associated CRC versus UC-associated CRC remain largely unexplored. Future spatial transcriptomic studies comparing these two backgrounds are warranted to refine precision stromal targeting.

While clinical manifestations of fibrosis differ, human studies on IBD-CRC often utilize cohorts from 'mixed IBD' populations due to the shared endpoint of inflammation-driven malignancy. However, it is plausible that the pre-malignant stromal 'soil' reflects the underlying disease-specific fibrotic program. For instance, CD-derived fibroblasts, exposed to transmural injury, may exhibit a more aggressive myofibroblastic phenotype characterized by higher contractility and a unique secretome compared to UC-derived fibroblasts, which are shaped by chronic mucosal erosion. Future research distinguishing these two backgrounds is essential to determine if the resulting CAFs retain disease-specific 'epigenetic scars' that influence tumor progression.

### From chronic colitis to premalignant niche: stromal priming for carcinogenesis

The transition from chronic inflammation to malignancy is driven by a fundamentally remodeled "pre-malignant niche." Persistent epithelial barrier disruption—often triggered by the loss of apical polarity regulators like aPKC λ/ι [25,26] —leads to dysbiosis and chronic microbial sensing [13,40] . This environment forces resident fibroblasts to undergo a permanent shift. Key drivers such as hypoxia and ECM stiffening via LOX upregulation physically strain the epithelium, while metabolic enzymes like PFKFB3 and cytokines like OSM create a self-sustaining inflammatory loop [6,8,19,23]. These factors collectively induce DNA damage [41], transform the stem cell niche [29], and initiate "immunological exhaustion" long before overt dysplasia appears. This "primed soil" ensures that once an oncogenic hit occurs, the microenvironment is already optimized for rapid tumor expansion and immune evasion.

## Roles of fibroblasts in IBD-associated colorectal cancer

**Fig. 2:**
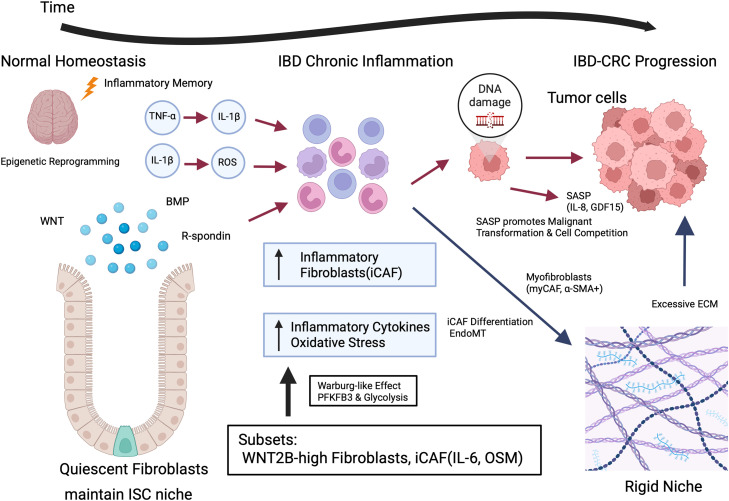
Schematic representation of the stromal evolution from intestinal homeostasis to IBD-associated colorectal cancer (IBD-CRC). The progression from chronic inflammation to malignancy is orchestrated by a dynamic transformation of the intestinal stromal compartment. Normal Homeostasis (Left): Under physiological conditions, quiescent fibroblasts maintain the intestinal stem cell (ISC) niche through the secretion of essential morphogens such as WNT, BMP, and R-spondin. IBD Chronic Inflammation (Center): Persistent exposure to inflammatory mediators (e.g., TNF-α, IL-1β, and ROS) triggers an "inflammatory memory" characterized by epigenetic reprogramming and a sustained iCAF (inflammatory Cancer-Associated Fibroblast) state. This phase is marked by a significant metabolic shift toward enhanced glycolysis (Warburg-like effect), mediated by the upregulation of PFKFB3. Activated fibroblasts form inflammatory cell aggregates and engage in bidirectional crosstalk with immune cells, further amplifying the inflammatory loop. IBD-CRC Progression (Right): Chronic inflammation and persistent iCAF activity lead to DNA damage in the epithelium and the accumulation of senescent fibroblasts. These senescent cells exhibit a Senescence-Associated Secretory Phenotype (SASP), secreting factors such as IL-8 and GDF15 that promote malignant transformation and cell competition. Stromal Remodeling and Niche Construction (Bottom): Throughout the transition, iCAFs differentiate into myCAFs through processes such as EndoMT, leading to excessive ECM deposition. This results in the formation of a "Rigid Niche" characterized by increased tissue stiffness, which further drives tumor invasion and progression.

IBD-CRC is a specific type of colorectal cancer that develops against a background of chronic inflammation and exhibits a distinct molecular pathology compared to sporadic colorectal cancer. In this complex process of carcinogenesis, fibroblasts dramatically transform their function, becoming Cancer-Associated Fibroblasts (CAFs) that are deeply involved in the formation of the Tumor Microenvironment (TME) and the multi-step progression of carcinogenesis (Fig. 2). It is important to acknowledge that much of our current understanding of CAF biology is derived from studies on sporadic colorectal cancer or other solid tumors. In sporadic CRC, CAFs are often viewed as a "reactive" stroma emerging in response to epithelial mutations such as *APC* loss [42]. However, in the context of IBD-CRC, CAFs evolve within a "primed" inflammatory environment [9]. While fundamental CAF functions—such as ECM remodeling and growth factor secretion—are likely shared across cancer types, the pre-neoplastic activation driven by chronic cytokine exposure and microbial sensing is a unique hallmark of IBD-associated carcinogenesis [[1], [2], [3]]. Throughout this review, we distinguish between these established general CAF mechanisms and those specifically validated in IBD models.

### The concept of CAFs in IBD-CRC: from "Reactive" to "Primed" stroma

Specifically, early epithelial signaling defects, such as the loss of PKC λ/ι, trigger Paneth cell disruption and dysbiosis, which in turn activates resident fibroblasts through chronic microbial sensing [25,26]. Thus, in IBD-CRC, the activated stroma acts not just as a supporter but as an initiator of the neoplastic sequence, following a "soil-corrupting-seed" dynamic. However, the proposal of fibroblasts as primary 'TME engineers' must be viewed within the context of stromal complexity. The transition from inflammation to malignancy is likely not driven by a single cell type but by a coordinated co-evolutionary process involving epithelial clones, infiltrating immune cells, and diverse stromal subpopulations. Therefore, the 'engineering' capacity of fibroblasts should be interpreted as their ability to sense and amplify microenvironmental cues, rather than acting as the sole determinants of the neoplastic niche.

### Diversity of CAF subsets and the serrated evolution

The advancement of single-cell technologies has revealed that CAFs in IBD-CRC are highly heterogeneous, comprising subsets such as myCAFs, iCAFs, apCAFs, and senCAFs [10,11,43]. Notably, the molecular evolution of these subsets is closely linked to the stepwise loss of aPKC isoforms. While epithelial PKC λ/ι deficiency creates the pro-inflammatory "soil" characteristic of IBD, the subsequent loss of PKC λ/ι acts as a critical switch that drives the progression toward serrated adenoma-adenocarcinoma [6,26]. This genetic synergy uniquely shapes the CAF landscape, enriching for subsets that support the mesenchymal and aggressive features of serrated-track IBD-CRC (Table 1). For instance, the MYH11+ myCAF subset, prevalent in the distal colon, contributes to the high degree of desmoplasia and clinical aggressiveness typical of IBD-associated lesions [44]. At the single-cell level, while subsets like iCAFs and myCAFs are identified across various IBD environments, their relative abundance may vary between CD-associated and UC-associated CRC. Given that CD is characterized by deeper mesenchymal involvement, the myCAF population in CD-CRC might be more predominant or spatially organized differently compared to UC—CRC, where the inflammatory infiltrate is more superficial. Identifying these subtle shifts is critical for developing precision stromal therapies tailored to the patient’s primary IBD diagnosis.

**Table 1:** Major CAF Subtypes and Their Roles in IBD-CRC.

**CAF Subtype**	**Representative Markers**	**Primary Functions (IBD/IBD-CRC)**	**Associated Pathways**	**Clinical Significance**
**Myofibroblastic CAF (myCAF)**	α-SMA, ACTA2, MYH11 (L-CRC specific)	Excessive ECM production, fibrosis (stricture), promotion of tumor invasion. Immune suppression (Treg induction).	TGF-β/Smad, PDGF, Wnt/β-catenin	Marker for intestinal stricture and poor prognosis in L-CRC; Major contributor to CMS4 mesenchymal signature.
**Inflammatory CAF (iCAF)**	PDPN, IL-6, IL-1β, CXCL12	Maintenance of chronic inflammation, recruitment of immune cells (TAMs, Tregs), secretion of inflammatory mediators.	NF-κB, STAT3, PFKFB3 (Enhanced glycolysis)	Amplification of chronic inflammation; target for metabolic therapies; fuels the inflammatory niche of CMS4 CRC.
**Antigen-Presenting CAF (apCAF)**	MHC Class II, CD74, Cathepsin S	Induction of T-cell exhaustion, immune evasion via cross-presentation of neoantigens.	T-cell exhaustion pathways, Cathepsin S-dependent presentation	Contributor to resistance against immune checkpoint inhibitors (ICIs).
**Senescent CAF (senCAF)**	p16, β-galactosidase, GDF15	Secretion of SASP factors, initiation of carcinogenesis, promotion of EMT.	DDR pathways, NF-κB/IL-6 axis	Target for Senolytics.

### Inflammatory memory: metabolic and epigenetic imprinting

The persistence of activated CAFs, even during clinical remission, has been proposed to be driven by "inflammatory memory" encoded within the stroma [29]. This conceptual framework suggests that prior inflammatory exposure induces long-lasting functional changes in fibroblasts.

One key feature associated with this phenomenon is a metabolic shift toward glycolysis, characterized by the upregulation of PFKFB3 [8]. This glycolytic reprogramming has been primarily demonstrated in murine colitis models and in vitro activated fibroblasts. While single-cell analyses of human IBD-CRC samples indicate similar metabolic shifts, further validation in patient-derived primary cells is required to confirm the persistence of this "metabolic memory" in the human disease context [29].

These stable modifications are thought to maintain the stroma in a pro-tumorigenic state, potentially providing a "Warburg-like" metabolic environment that may support epithelial transformation and contribute to the early stages of malignancy. However, direct evidence demonstrating the long-term persistence of this phenomenon in human tissues remains limited.

### Stromal Senescence and SASP: Sustaining the Pre-malignant niche

In the context of chronic inflammation, fibroblasts undergo cellular senescence induced by persistent DNA damage and oxidative stress [42,45]. Although many SASP-related mechanisms have been extrapolated from sporadic cancer models or general aging studies, their role in IBD-CRC is increasingly supported by evidence from chronic colitis-associated murine models. In these experimental systems, microbial by-products further amplify the SASP via TLR signaling. This continuous secretome paracrinely induces epithelial-mesenchymal transition (EMT) and genomic instability, representing a potential driver of the dysplasia-carcinoma sequence specifically within the inflammatory niche.

### Orchestrating Immune evasion: the convergence of stromal signaling

While the precise orchestrators of immune evasion in IBD-CRC are still being elucidated, several mechanisms can be extrapolated from sporadic CRC and other TMEs where stromal-mediated immunosuppression is well-documented. In IBD-CRC, universal stromal barriers are further complicated by the underlying chronic inflammatory infiltrate, potentially creating an even higher threshold for effective anti-tumor immunity. The convergence of metabolic reprogramming, persistent SASP, and niche stiffening culminates in a highly immunosuppressive TME, which constitutes a critical barrier to anti-tumor immunity in IBD-CRC. The various stromal mechanisms—metabolic shifts, SASP, and subset diversification—converge to orchestrate a profoundly immunosuppressive TME. apCAFs directly induce T cell exhaustion by cross-presenting tumor antigens via the MHC II-Cathepsin S pathway [46,47] . Simultaneously, CAF-derived CXCL1 and CXCL8 recruit CXCR2-positive Myeloid-Derived Suppressor Cells (MDSCs), creating an immunological "shield" around the tumor [[48], [49], [50]]. Furthermore, stromal-derived IL-6 and TGF-βpromote Treg plasticity, converting stable Tregs into pathogenic Th17-like cells [51].

### Metabolic symbiosis and EV-mediated progression: driving resistance

**Fig. 3:**
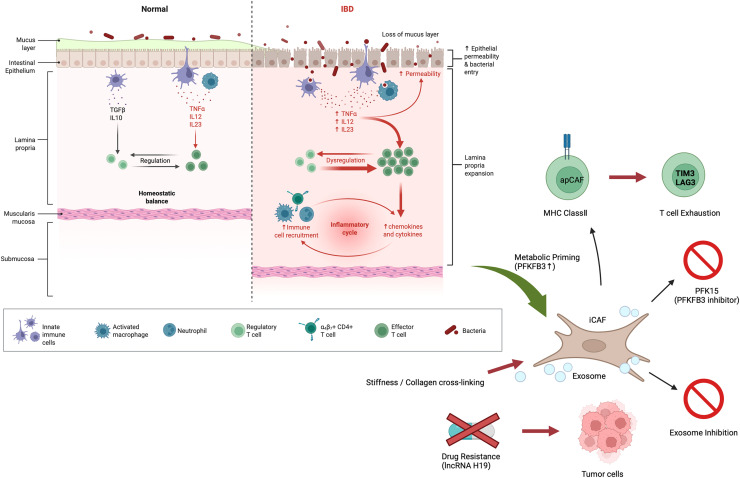
Stromal Evolution and Functional Reprogramming in IBD-Associated Colorectal Cancer (IBD-CRC). The schematic illustrates the transition from chronic intestinal inflammation to a pro-tumorigenic microenvironment orchestrated by Cancer-Associated Fibroblast (CAF) subsets. IBD Inflammatory Environment (The "Corrupt Soil"): Persistent inflammation in IBD leads to the loss of the mucus layer, increased epithelial permeability, and dysregulated recruitment of immune cells (e.g., neutrophils, activated macrophages). This environment, rich in pro-inflammatory cytokines such as TNF-α, IL-12, and IL-23, triggers "Metabolic Priming" and "Inflammatory Memory" in resident fibroblasts. CAF Subset Differentiation and Metabolic Reprogramming: Activated fibroblasts differentiate into distinct functional subsets, including iCAFs and apCAFs. This process is characterized by a glycolytic shift (Metabolic Priming) mediated by the upregulation of PFKFB3. Physical remodeling, including increased tissue stiffness and collagen cross-linking (LOX activity), further reinforces this stromal activation. Immune Evasion and T-cell Exhaustion: The apCAF subset directly interacts with CD8+ T cells via MHC Class II, inducing a state of "T-cell Exhaustion" marked by the expression of inhibitory checkpoints such as TIM3 and LAG3. Exosome-Mediated Drug Resistance: CAFs secrete exosomes containing bioactive molecules, such as lncRNA H19, which are transferred to tumor cells to promote stemness and resistance to chemotherapeutic agents like Oxaliplatin. Therapeutic Targets: Potential intervention points include the inhibition of the glycolytic enzyme PFKFB3 using PFK15, and the blockade of CAF-derived exosome release or uptake to restore anti-tumor immunity and drug sensitivity.

As the tumor progresses, CAFs and malignant cells establish a metabolic symbiosis known as the "Reverse Warburg Effect". In vitro co-culture experiments using sporadic CRC cell lines and fibroblasts have shown that CAFs supply lactate and glutamine to tumor cells, supporting their rapid proliferation and survival under nutrient-deprived conditions (Fig. 3). This interaction is further mediated by Extracellular Vesicles (EVs) and exosomes, which transfer non-coding RNAs [52,53]. While these exosome-mediated pathways are well-documented in general colorectal malignancy, their specific contribution to chemoresistance in human IBD-CRC patients remains an area of ongoing clinical investigation. These exosomal cargos activate the PI3K/AKT and Wnt/β-catenin pathways, driving the acquisition of stemness and potent resistance to chemotherapies such as 5-FU and oxaliplatin [[54], [55], [56]]. Thus, the co-evolution of the stroma and epithelium leads to a high-threshold therapeutic resistance that is a hallmark of advanced IBD-CRC.

## Interaction CAFs in TME

### Role of CAFs and intestinal myofibroblasts in the early stages of IBD-associated carcinogenesis

CAFs promote the formation of the TME and carcinogenesis by playing a multifaceted role in facilitating tumor cell proliferation, survival, invasion, metastasis, and even the acquisition of drug resistance [[57], [58], [59], [60]]. The diverse immune mediators, as well as the abundance and activation status of immune cells in the local microenvironment, determine the balance between the anti-tumor response and the tumor-promoting effects of inflammation [61]. The TME, formed by infiltrating immune cells, stromal cells, and tumor cells, is essential for sustaining proliferative signaling, resisting cell death, and inducing angiogenesis.

First, CAFs and their precursors, such as Intestinal Myofibroblasts (IMFs), are involved in the process of carcinogenesis from its initial stages. Chronic inflammation in IBD drives a multi-step carcinogenic process known as the dysplasia-carcinoma sequence, which encourages continuous damage and regeneration of epithelial cells. In this specific context, IMFs are not merely passive bystanders but active drivers of malignant transformation. Kawamura et al. highlighted that phenotypic changes in IMFs occur *prior* to the establishment of invasive cancer, specifically during the dysplastic phase [62]. Activated fibroblasts and IMFs, particularly in colitis-associated models mirroring UC progression, support the proliferation and survival of dysplastic epithelial cells through the secretion of inflammatory cytokines (IL-6, TNF-α), growth factors (HGF, IGF), and chemokines like CXCL12 [63,64]. In addition to these soluble factors, CAF-derived LOXL2 has been identified as a key stromal mediator that facilitates the transition from dysplasia to invasive carcinoma [65]. By catalyzing the cross-linking of collagen fibers, LOXL2 creates a stiffened scaffold that promotes integrin-mediated signaling and cellular motility, a mechanism that mirrors its role in other gastrointestinal cancers. Furthermore, CAFs strongly promote tumor cell proliferation and survival via the IL-6/STAT3 pathway, contributing to rapid tumor growth and apoptosis resistance [66].

Furthermore, recent spatial transcriptomics studies suggest that the early accumulation of CAFs at the crypt base in inflamed mucosa creates a "pro-tumorigenic niche". Consistent with the paradigm that SASP-expressing stromal cells facilitate the malignant transformation of neighboring pre-neoplastic cells [67], the persistent inflammatory signaling in IBD-derived fibroblasts may act as an extrinsic driver of epithelial mutations and clonal expansion. Within this niche, CAFs overactivate Wnt signaling in stem cells, accelerating the transition from dysplasia to invasive carcinoma [68]. Parallel to these epithelial changes, the upregulation of SOX2 in the stromal compartment further orchestrates the development of a pro-tumorigenic niche. Stromal SOX2 induces the generation of SFRP1/2-expressing CAFs, which modulate the local Wnt signaling environment to support the survival and expansion of dysplastic cells, representing another pivotal molecular axis in inflammation-driven colorectal carcinogenesis [69]. Beyond local progression, the TGF-β-mediated transformation of mesenchymal cells contributes to the pre-metastatic niche formation. Specifically, the CXCR4/TGF-β1 axis has been shown to drive the differentiation of resident mesenchymal cells into CAF-like phenotypes, thereby promoting the liver metastasis of colorectal cancer [20].

### Angiogenesis and Tenascin-C in colitis-associated cancer

Sufficient blood supply, or angiogenesis, is essential for tumor growth and metastasis. In the context of IBD, where the mucosal barrier is repeatedly breached and repaired, the regulation of angiogenesis is distinct from sporadic colorectal cancer. While CAFs typically produce pro-angiogenic factors such as VEGF and bFGF [70], recent studies clarify a unique mechanism driven by IMFs in the development of IBD-CRC.

Kawamura et al. identified Tenascin-C (TNC) as a critical stromal factor that links chronic inflammation to carcinogenesis [62]. Through comprehensive transcriptome analysis comparing normal fibroblasts and IMFs, TNC was found to be one of the most significantly upregulated genes in the IBD-CRC microenvironment. Crucially, TNC expression is minimal in normal mucosa and non-dysplastic inflammatory tissue but is markedly upregulated specifically in the stroma surrounding dysplastic lesions [62]. This suggests that TNC upregulation in IMFs is a pivotal event in the early "dysplasia-carcinoma sequence," serving as a potential biomarker for high-risk lesions in IBD patients. Mechanistically, TNC produced by IMFs does not merely provide structural support; it actively promotes angiogenesis by interacting with integrin αvβ3 on endothelial cells. Immunohistochemical analysis revealed that TNC physically surrounds and encloses integrin-positive microvessels within the dysplastic stroma, creating a niche that fosters neovascularization. This interaction is functionally significant: the administration of ATN-161, a specific antagonist of integrin αvβ3, significantly suppressed tumorigenesis in AOM/DSS mouse models by inhibiting angiogenesis [62]. Consequently, targeting the IMF-TNC-Integrin αvβ3 axis represents a novel and promising therapeutic strategy for preventing IBD-CRC. Beyond TNC, CAFs in IBD-CRC contribute to pathological angiogenesis through ECM stiffness-induced mechanotransduction. The dense collagen cross-linking by LOX—upregulated in the IBD stroma—activates the YAP/TAZ signaling pathway in both CAFs and endothelial cells. This mechanical signaling induces the expression of pro-angiogenic genes independent of VEGF, suggesting why anti-VEGF therapies often show limited efficacy in highly fibrotic CRC [24]. Importantly, hypoxia-induced LOX does not merely act as a structural cross-linker; it functions as a critical biochemical signal that triggers the epithelial-mesenchymal transition (EMT) [23] . By remodeling the collagen network and activating intracellular signaling pathways, LOX enhances the motility and invasiveness of pre-neoplastic cells, thereby accelerating the progression from dysplasia to invasive carcinoma. Furthermore, CAFs secrete Angiopoietin-like 4 (ANGPTL4), which destabilizes endothelial junctions, facilitating both nutrient supply to the growing tumor and early hematogenous metastasis [71].

### Fibroblast-Macrophage crosstalk: orchestrating Pro-Tumor inflammation

While Tumor-Associated Macrophages (TAMs) are the primary effectors of inflammation-associated carcinogenesis, recent evidence suggests that their recruitment and functional polarization are strictly dictated by the stromal compartment. In the IBD microenvironment, activated Intestinal Myofibroblasts (IMFs) and CAFs serve as the "command center" for myeloid cell dynamics.

First, activated fibroblasts constitute the major source of monocyte-attracting chemokines, including CCL2 (MCP-1) and CSF1 (M-CSF). Through the secretion of these factors, fibroblasts actively recruit circulating monocytes into the inflamed mucosa and the TME, creating a niche rich in myeloid cells. Once recruited, the functional fate of these macrophages is molded by fibroblast-derived signals. Unlike the acute inflammatory response, CAFs in the TME secrete high levels of IL-6, GM-CSF, and lactate, which skew macrophage polarization toward an immunosuppressive and pro-tumorigenic M2-like phenotype rather than the anti-tumor M1 phenotype.

Crucially, this interaction forms a self-amplifying paracrine loop. Macrophage-derived IL-1β and TNF-α act on fibroblasts to induce the expression of COX-2, leading to the massive release of Prostaglandin E2 (PGE2). This stromal PGE2, in turn, suppresses the cytotoxic activity of immune cells and further reinforces the M2 polarization of TAMs [72]. It is within this fibroblast-instructed microenvironment that TAMs sustain the production of IL-23, the master regulator of the IL-23/IL-17 axis, thereby driving the progression from colitis to cancer. Thus, fibroblasts are not merely bystanders but are the essential engineers that maintain the "chronic" nature of inflammation required for carcinogenesis. Recent single-cell studies have identified a specific subset of SPP1+ macrophages that co-localize with CAFs in the IBD-CRC TME. This interaction is mediated by the CXCL12-CXCR4 axis, where CAF-derived CXCL12 recruits monocytes and polarizes them into SPP1+ TAMs. These macrophages, in turn, produce TGF-β, forming a reciprocal feedback loop that further activates fibroblasts into myofibroblastic CAFs (myCAFs), leading to profound desmoplasia and immune exclusion [73].

## Molecular-driven therapeutic strategies: targeting the stromal axis

It should be noted that while the molecular understanding of the stromal axis has advanced rapidly, most stromal-targeted therapeutic strategies specifically for IBD-CRC remain in the preclinical stage. Currently, no stromal-specific agents have been approved specifically for the prevention or treatment of colitis-associated malignancy in clinical practice. However, several agents targeting similar pathways in sporadic colorectal cancer or other fibrotic diseases are under clinical investigation, offering significant potential for drug repositioning. The following sections delineate these emerging preclinical concepts and their translational prospects. The transition from chronic inflammation to IBD-CRC is orchestrated by a molecularly distinct population of fibroblasts, making them prime targets for therapeutic intervention. Rather than broad stromal depletion, which has historically shown paradoxical tumor-promoting effects, modern strategies focus on "stromal reprogramming" or targeting specific molecular vulnerabilities within CAF subsets. Clinical evidence indicates that the OSM-OSMR axis in fibroblasts mediates resistance to anti-TNF therapies [6]. Therefore, targeting the fibroblast-driven 'alternative inflammatory pathways' and reversing the mechanical stiffness of the stroma is not merely a supplementary approach, but a fundamental requirement to achieve complete mucosal healing and prevent the transition to IBD-CRC(Table 2).

**Table 2:** Stromal Cell Mechanisms Driving the Transition from IBD to IBD-CRC.

**Progression Stage**	**Mechanism**	**Role of Stromal Cells**	**Key Molecules & Mediators**	**Therapeutic Targets**
IBD Phase(Chronic Inflammation)	Inflammatory Memory & Epigenetics	Permanent maintenance of inflammatory response and priming.	Histone modifying enzymes, WNT2B (high), Chromatin remodeling	Epigenetic editors / modifiers
IBD / Pre-Cancer Phase	Epithelial-Stromal Crosstalk	Hyaluronan-driven remodeling & priming of the niche.	aPKC deficiency, Hyaluronan, CD44	Hyaluronan inhibitors, CD44 antagonists
Pre-Cancer / Early Cancer	Cellular Senescence & SASP	Paracrine induction of proliferation via SASP factors.	SASP factors (IL-8, HGF, GDF15)	Senolytics, Senomorphics (SASP inhibitors)
IBD-CRC Phase(Invasion / Drug Resistance)	Exosome-Mediated Transfer	Conferring malignant phenotypes (stemness, chemoresistance).	Exosomal ncRNAs (lncRNA H19, circSLC7A6)	EV release/uptake inhibitors

### Prevention of malignant transformation by targeting inflammatory fibroblasts

Since fibroblasts act as initiators of the neoplastic sequence, targeting them during the chronic inflammatory phase represents a powerful preventive strategy. Preclinical studies have shown that the genetic or pharmacological inhibition of fibroblast-specific signaling can attenuate colitis severity and subsequent tumor development. For example, targeting the IL-11/GP130 axis or STAT3 signaling specifically in fibroblasts has been shown to reduce mucosal damage and suppress the formation of AOM/DSS-induced tumors [66]. Additionally, as mentioned previously, inhibition of PFKFB3 in fibroblasts not only reduces fibrotic markers but also significantly dampens the inflammatory infiltrate in DSS-induced colitis models [8]. These findings suggest that "stromal-targeted prophylaxis" could potentially extend the period of clinical remission and block the transition to IBD-CRC.

### Direct depletion and reprogramming of CAF subsets

Current research is shifting from non-specific myofibroblast targeting to the selective inhibition of pro-tumorigenic CAF populations. Fibroblast Activation Protein (FAP) remains a primary target; however, next-generation FAP-targeted chimeric antigen receptor (CAR) T-cells and FAP-activated prodrugs (e.g., AVA6000) are being developed to minimize systemic toxicity [74]. Furthermore, the TGF-β/Smad signaling pathway, a master regulator of CAF activation, is being targeted with small molecule inhibitors and neutralizing antibodies (e.g., Vactosertib) to revert CAFs to a more quiescent, "normal-like" state, thereby reducing ECM stiffness and improving drug delivery [75]. Furthermore, targeting CAF-derived proteins that orchestrate immune exclusion, such as TSP2, represents a promising strategy to overcome resistance to immune checkpoint inhibitors (ICIs); disrupting the TSP2-mediated stromal barrier could potentially reprogram the 'cold' TME of IBD-CRC into an 'immunologically hot' environment, thereby sensitizing tumors to PD-1/PD-L1 blockade.

### Targeting metabolic vulnerabilities

As previously discussed, the glycolytic shift (Warburg effect) in IBD-CAFs is a hallmark of their activation. Inhibiting PFKFB3 with small molecules like PFK158 has shown promise in preclinical models by suppressing the secretion of pro-inflammatory cytokines and reducing the stromal support for tumor cells [8,76]. Additionally, targeting the monocarboxylate transporters (MCT1/4), which facilitate the "Reverse Warburg Effect" by transporting lactate from CAFs to tumor cells, provides a metabolic means to "starve" the malignancy [77]. Given that hypoxia simultaneously drives metabolic reprogramming and LOX-mediated EMT, dual targeting of glycolytic enzymes like PFKFB3 and hypoxic mediators such as LOX may offer a synergistic approach to disrupt the pro-tumorigenic niche in IBD-CRC [23].

### Modulating the Secretome: Senomorphics and exosome inhibitors

The Senescence-Associated Secretory Phenotype (SASP) in the IBD stroma acts as a continuous source of oncogenic stimuli [54,78]. "Senomorphic" agents, such as JAK inhibitors (Ruxolitinib) or rapamycin (mTOR inhibitors), can suppress the production of GDF15, IL-8, and HGF without inducing cell death, effectively neutralizing the pro-carcinogenic "soil." Furthermore, blocking the biogenesis or uptake of CAF-derived exosomes—specifically those carrying chemoresistance-inducing lncRNAs like H19—represents a novel approach to overcoming 5-FU and Oxaliplatin resistance in IBD-CRC.

### Erasing "inflammatory memory" via epigenetic modifiers

The persistence of activated CAFs even after inflammatory remission suggests a stable epigenetic "imprint." Targeting chromatin remodelers, such as BET bromodomain proteins (BRD4) or Histone Deacetylases (HDACs), offers a strategy to "reset" the stromal cell state. Inhibitors like JQ1 have demonstrated the ability to suppress the pro-inflammatory and pro-fibrotic signature of IBD-derived fibroblasts, potentially preventing the initiation of the dysplasia-carcinoma sequence [29,79]. Recent studies have further elucidated the specific role of BRD4 as a master regulator of the inflammatory cytokine response in the intestinal mucosa. BRD4 functions by recruiting the transcriptional machinery to the promoters of pro-inflammatory genes, and its inhibition has been shown to markedly suppress the expression of key cytokines involved in IBD pathogenesis [80]. Notably, BRD4 has been identified as a positive regulator of IL-34 production in the gut; pharmacological blockade of BRD4 significantly reduces IL-34 levels in both intestinal transition and established IBD-CRC environments [81]. This molecular link between epigenetic readers (BRD4) and stromal-derived cytokines (IL-34) underscores the potential of BET inhibitors not only in "resetting" the fibroblast identity but also in dismantling the cytokine-driven networks that sustain the pre-malignant niche. Given the critical role of LOX family members, particularly LOXL2, in both fibrosis and EMT, the development of specific LOXL2 inhibitors or monoclonal antibodies represents a promising therapeutic avenue to reprogram the IBD-CRC stroma and block the progression of inflammation-driven malignancy [65].

### Challenges and dual roles of CAFs in therapeutic targeting

While targeting CAFs offers a promising therapeutic avenue, it is essential to consider the ongoing controversies and the functional duality of the stroma. Early attempts at indiscriminate stromal depletion in other malignancies, such as pancreatic cancer, unexpectedly led to more aggressive tumor phenotypes and reduced survival, suggesting that certain CAF subsets may exert a "tumor-restraining" rather than "tumor-promoting" effect [82]. In the context of IBD-CRC, the stroma may similarly play a role in tumor containment or wound healing during clinical remission. Therefore, the historical paradigm of broad stromal depletion is shifting toward "stromal reprogramming" or subset-specific targeting. Distinguishing between pro-tumorigenic subsets (e.g., iCAFs and FAP+ myCAFs) and potentially protective populations is a critical prerequisite for the clinical success of any stromal-targeted intervention. Future strategies must aim to neutralize the pathogenic secretome while preserving the structural integrity and homeostatic functions of the intestinal stroma.

## Conclusion and future perspectives

In the initiation and progression of IBD-CRC, fibroblasts shift their role from passive scaffold cells to dynamic orchestrators of the TME, actively participating in multiple hallmarks of cancer, including the maintenance of chronic inflammation, fibrosis, immunosuppression, and the acquisition of drug resistance. As outlined in this review, the induction of T cell exhaustion by AP-CAFs identified through scRNA-seq, metabolic reprogramming via PFKFB3, and paracrine signaling mediated by SASP and exosomal ncRNAs are key molecular mechanisms crucial for deeply understanding IBD-CRC pathogenesis. Future research must bridge these sophisticated molecular mechanisms to clinical application. Specifically, there is an urgent need for the comprehensive elucidation of the epigenetic pathways driving CAF origin and differentiation, the development of highly selective molecular agents that target only specific pro-tumorigenic CAF subtypes, and the clinical evaluation of combination therapies-combining FAP inhibitors or metabolic inhibitors with existing chemotherapy and immune checkpoint inhibitors. Controlling the dynamics of fibroblasts represents one of the most promising strategies for innovating the treatment paradigm for IBD-CRC and improving patient prognosis. The concept of 'erasing' inflammatory memory through epigenetic modifiers (e.g., HDAC inhibitors or bromodomain inhibitors) represents a paradigm shift from passive symptom management to active stromal reprogramming. Integrating the findings on epithelial regulators like PKC λ/ι, which control Paneth cell fate and microbial homeostasis, with our understanding of fibroblast dynamics will be crucial. Future strategies should aim to prevent the 'corrupt soil' formation by stabilizing both epithelial barrier functions and stromal phenotypes.

## Funding sources

This research did not receive any specific grant from funding agencies in the public, commercial, or not-for-profit sectors.

## Declaration of generative AI use

During the preparation of this work the authors used Gemini (Google) in order to improve the English phrasing. After using this tool/service, the authors reviewed and edited the content as needed and take full responsibility for the content of the publication.

## CRediT authorship contribution statement

**Nobuhiro Naito:** Writing – review & editing, Writing – original draft, Visualization, Validation, Software, Project administration, Formal analysis, Data curation. **Hiroaki Kasashima:** Writing – review & editing, Project administration, Funding acquisition, Conceptualization. **Tatsunari Fukuoka:** Visualization. **Yuki Nakanishi:** Writing – review & editing, Visualization, Project administration. **Naoko Ohtani:** Writing – review & editing, Visualization, Project administration. **Kiyoshi Maeda:** Supervision.

## Declaration of competing interest

The authors declare that they have no known competing financial interests or personal relationships that could have appeared to influence the work reported in this paper.
